# Prevalence of polymorphisms in the ANKK1, DRD2, DRD3 genes and metabolic syndrome in refractory schizophrenia

**DOI:** 10.1590/1518-8345.2222.2983

**Published:** 2018-05-17

**Authors:** Jeizziani Aparecida Ferreira Pinto, Pedro Henrique Batista de Freitas, Fernanda Daniela Dorneles Nunes, Paulo Afonso Granjeiro, Luciana Lara dos Santos, Richardson Miranda Machado

**Affiliations:** 1 MSc in Nursing.; 2 MSc, in Nursing, RN, Family Health Strategy, Secretaria Municipal de Saúde, Divinópolis, MG, Brazil.; 3 Graduated, MSc in Nursing, Graduate Program in Nursing, Universidade Federal de São João Del Rei, Divinópolis, MG, Brazil, Scientific Initiation Scholarship.; 4 PhD, Functional and Molecular Biology, Adjunct Professor, Pharmacy, Universidade Federal de São João Del Rei, Divinópolis, Minas Gerais, Brazil.; 5 PhD, Genetic, Adjunct Professor, Biological Sciences, Universidade Federal de São João Del Rei, Divinópolis, Minas Gerais, Brazil.; 6 PhD, Psychiatry, Adjunct Professor, Nursing, Universidade Federal de São João Del Rei, Divinópolis, Minas Gerais, Brazil.

**Keywords:** Nursing, Schizophrenia, Genetic Polymorphism, Metabolic X Syndrome, Clozapine, Health Profile

## Abstract

**Objective:**

to estimate the prevalence of TaqIA, -141C and rs6280 polymorphisms of the ANKK1, DRD2 and DRD3 genes and evaluate their association with the occurrence of metabolic syndrome in patients with refractory schizophrenia.

**Method:**

cross-sectional study conducted in the Extended Western Region of Minas Gerais, with refractory schizophrenic patients using the antipsychotic clozapine. Sociodemographic, clinical, anthropometric, biochemical and genetic data were collected. Univariate analysis of the data was performed.

**Results:**

seventy-two patients participated in the study and the occurrence of Metabolic Syndrome was observed in 47.2% of them. There was no association between Metabolic Syndrome and the studied polymorphisms. There was a statistically significant difference in the low HDL parameter with homozygous genotype for the C allele of the -141C polymorphism of the DRD2 gene.

**Conclusion:**

a high prevalence of MS was evidenced. The -141C polymorphism was associated with low HDL. Genetic analysis and identification of metabolic alterations in this group of patients can guide drug treatment and provide a better quality of life.

## Introduction

Schizophrenia is considered one of the most serious mental disorders nowadays, since its progression affects the quality of life of individuals who experience the disease and also of their relatives^1^. Schizophrenia affects more than 21 million people worldwide and is considered a major public health problem^1^ ^-^ ^2^. This disease is manifested mainly by the presence of positive symptoms (change in the thinking process, perceptions and affection) and negative symptoms (affective-volitional blunting, cognitive losses and depressive symptoms)^2^.

Approximately 40% of people with schizophrenia do not present adequate response to drug treatment and persist with symptoms of the disease; they are known as refractory schizophrenic people^1^ ^-^ ^3^. Although there is no single, globally accepted consensus, refractory schizophrenia can be characterized by partial response, for at least five years, to three different types of antipsychotics (at least two with different chemical structures); intolerance to adverse effects; and relapses or symptomatic deterioration despite the use of appropriate doses of the drugs^3^ ^-^ ^4^. The atypical antipsychotic clozapine is considered the gold standard for the drug treatment of refractory schizophrenia, being associated with clinical improvement and decreased hospitalizations^4^ ^-^ ^5^.

In this context, studies have indicated that genetic polymorphisms of dopaminergic pathways are related to etiopathogenesis and drug response to treatment, as well as to a greater susceptibility to clinical alterations. Unique or Single Nucleotide Polymorphisms (SNPs) consist in a variation of the DNA sequence caused by a single nucleotide exchange in the genome sequence. Some SNPs alter the amino acid composition of the protein and the expression of the receptor, which may have an effect on the phenotype of an individual, and can also serve as molecular markers of predisposition to certain types of diseases. The ability to detect polymorphisms at the DNA level is the basis of several studies that aim to identify whether this variation causes or contributes to a specific phenotype^5^ ^-^ ^6^.

Among the genetic polymorphisms of the most studied dopaminergic pathway are the *TaqIA, -141C* and *rs6280*. These variants are located in the *ANKK1, DRD2* and *DRD3* genes, respectively, which have already been addressed by other authors in the literature, relating them to the response to antipsychotics and metabolic alterations of the individuals making use of this drug treatment^4^ ^-^ ^5^. However, further in-depth analyses of how these variants in the DNA sequence can influence metabolic alterations in the carriers of these polymorphisms are necessary, as this theme is still little elucidated^7^.

The abovementioned polymorphisms occur in genes encoding proteins that contribute to adequate signaling of neurotransmitters in the mesocorticolimbic pathway or ventral tegmental area. These regions play an important role in motivation, guided thinking, affective balance and in the reward system, the latter being responsible for the feeling of satiety and appetite. Psychotropic drugs act primarily in these regions by lowering dopamine (DA) levels and therefore reducing psychotic symptoms. However, at very low levels, they can lead to cognitive impoverishment, depression and metabolic alterations^8^.

One of the potential consequences of this biochemical process is a condition known as Metabolic Syndrome (MS)^6^ ^-^ ^9^. MS is characterized by a set of risk factors that include abdominal obesity, insulin resistance, dyslipidemia and hypertension. The prevalence of MS in patients with refractory schizophrenia compared to patients with other forms of schizophrenia is higher and has more drastic repercussions, reaching 69%^9^. Although the causal relationship between refractory schizophrenia and MS is not fully understood, there is evidence of the association between second-generation antipsychotics and the development of this syndrome. It has been suggested that the presence of negative symptoms may increase the risk, which may be associated with the sedentary lifestyle and the poor quality of life resulting from this condition^10^ ^-^ ^11^.

The genetic analysis of refractory schizophrenic patients can become an essential tool in the perspective of a care plan using genetic counseling, which can serve as a basis to guide the drug treatment and consequently provide a better quality of life for these notably more severe patients. In view of the above, the present study aims to estimate the prevalence of *TaqIA, -141C* and *rs6280* polymorphisms of the *ANKK1, DRD2* and *DRD3* genes and to evaluate their association with the occurrence of MS in refractory schizophrenic patients.

## Method

This is a cross-sectional and analytical study carried out in the Extended Western Region of Minas Gerais with patients diagnosed with refractory schizophrenia using the antipsychotic clozapine. The following inclusion criteria were observed: medical diagnosis of refractory schizophrenia with use of the atypical antipsychotic clozapine, male or female patients older than 18 years and with understanding capacity attested by the Mini-Mental State Examination (MMSE)^12^. Pregnant women, participants who were not fasting, and those who had any condition that could interfere with the collection and measurement of data, such as the presence of physical disability that could impair the measurement of anthropometric characteristics, were excluded. The need for fasting was due to the performance of laboratory tests to verify the presence of MS.

The sample calculation was performed using the OpenEpi version 3.03a, considering a population of 169 individuals for an expected proportion of the event of 50%, a significance level of 5% and a margin of error of 10%. A sample of approximately 62 individuals was estimated. The final sample consisted of 72 participants.

All individuals who made up the eligible population were previously invited to collect data by sending letters and making a telephone contact, when they received all the necessary guidelines for the research. Data were collected from December 2014 to June 2015.

Data were collected at the Psychosocial Care Center type III of the municipality-polo of the Great West Region in scheduled date and time. The sociodemographic and clinical profile of the subjects of this study was surveyed through a semi-structured questionnaire prepared by the authors, including the following data: gender, age, marital status, number of children, family income, schooling, and people with whom they reside. The following clinical data was surveyed: time of psychiatric treatment, previous psychiatric hospitalization, time of use of clozapine, number of consultations per year, clinical diseases, use of medications, smoking, alcohol use, physical activity, perception about the effect of the medication and satisfaction with health.

For biochemical and genetic analyses, venous blood samples were taken from the cubital vein of the forearm after a 12-hour fast. The analysis was done in the biochemistry and genetics laboratory of the Federal University of São João Del-Rei/Center - Dona Lindu West Campus.

In the biochemical evaluation, laboratory tests of fasting glycemia (FG), high-density lipoprotein-cholesterol (HDL-c) and triglycerides (TG) were performed. MS was determined according to the criteria of the National Cholesterol Education Program (NCEP) Adult Treatment Panel III (ATP-III), which defines the MS as the presence of three or more of the following risk factors: central obesity (abdominal circumference > 102cm in men or > 88cm in women); high blood pressure (> 130/85mmHg) or on antihypertensive treatment; hyperglycemia (fasting blood glucose > 100mg/dL) or treatment with hypoglycemic drugs; high triglyceride concentration (>150mg/dL) or use of medication to reduce it; low HDL-c (< 40mg/dL in men or < 50mg/dL in women) or on use of low HDL-c medication^11^.

The genetic analysis of peripheral blood samples consisted of five steps: the first one was the extraction of genomic DNA using the QIAamp Mini Blood Kit (Qiagen) according to the manufacturer’s protocol; the second step was the amplification ofthe DNA target sequences of each of the genes involved in the study using Polymerase Chain Reaction (PCR); then, each sample of PCR product was subjected to electrophoresis to confirm the amplification of the regions of interest; for genotyping, the *Restriction Fragment Length Polymorphism* (RFLP) technique was used; and, finally, the samples digested in the previous step were submitted to an 8% polyacrylamide gel for separation of DNA bands, which are expected and identified by their respective sizes (number of nitrogenous bases).

Data processing and analysis were performed in the *Statistical Package for Social Science* (SPSS), version 20.0. To describe the results, frequency distribution tables were used for categorical variables. In the analysis of categorical variables, the *Pearson* chi-square of and *Fisher’s* exact test were used for the univariate analysis to evaluate the MS factors associated to the polymorphisms. A *p-value* < 0.20 was adopted for inclusion of variables in the model. The adjusted *odds ratio* (OR) with a respective 95% confidence interval (95% CI) was estimated. The goodness of fit of the model was assessed using the *Hosmer-Lemeshow* test.

*Hardy-Weinberg* equilibrium was analyzed through the *Pearson* chi-square test, comparing the frequencies of the genotypes observed with those expected for this population. This calculation was performed using the *Hardy-Weinberg* equilibrium calculator software including analysis for ascertainment bias (13).

The study was approved by the Research Ethics Committee under Protocol nº 1,406,658, in compliance with the recommendations of Resolution 466/2012 of the National Health Council.

## Results

An unintentional finding of the study was that both genders were equally represented among the 72 patients with refractory schizophrenia in use of clozapine (50%). The average age of the participants was approximately 43 years.

It was verified that the prevalence of MS in the studied population was 47.2%, being more frequent among females (58.8%), but it was also observed that males also had a high incidence rate (41.2%). Patients in the age group below 40 years were those with the highest prevalence of MS (38.2%). Another characteristic identified was the time of treatment with clozapine, in which a prevalence of 65.6% of MS was found in those who had used this medication for more than 5 years.

Table 1 below shows the percentage distribution of the occurrence of each variant, detailed according to genotypic and allelic frequencies. The *Hardy-Weinberg* equilibrium, as tested through the chi-square value, found at each genotype frequency is shown in the table. No deviation was identified. The chi-square table was used as reference.

**Table 1:** Prevalence of the analyzed polymorphisms (n = 72). Extended Western Region of Minas Gerais, MG, Brazil, 2015

	Percentage	N	p-value^*^
*Taq*IA polymorphism			
Genotypes			
Homozygous for the C allele	51.4	37	0.0454
Heterozygous	41.7	30	
Homozygous for the T allele	6.9	5	
Alleles			
C	72.2	104	
T	28.0	40	
*-141C* polymorphism			
Genotypes			
Homozygous for the Del C allele	68.1	49	0.0000
Heterozygous	25.0	18	
Homozygous for the Ins C allele	6.9	5	
Alleles			
Del C	80.5	116	
Ins C	19.4	28	
*rs6280* polymorphism			
Genotypes			
Homozygous for the C allele	23.6	17	0.0032
Heterozygous	41.7	30	
Homozygous for the T allele	34.7	25	
Alleles			
C	55.5	80	
T	44.4	64	

N: observed frequencies * p-value: chi-square test

Table 2 shows the association of the studied polymorphisms with MS. Regarding genotypic and allelic analyses, there was no statistically significant difference with MS (p > 0.05).

**Table 2:** Analysis of association of polymorphisms in patients with and without metabolic syndrome* (n = 72). Extended Western Region of Minas Gerais, MG, Brazil, 2015

Polymorphism	Without MS*		With MS*		p^†^
	N	%	N	%	
*Taq*IA genotypes					
Homozygous for the C allele	21	55.3	16	47.1	0.708
Heterozygous	15	39.5	15	44.1	
Homozygous for the T allele	2	5.3	3	8.8	
*Taq*IA alleles					
C	36	67.9	31	63.3	0.620
T	17	32.1	18	36.7	
*-141C* genotypes					
Homozygous for the Del C allele	25	65.8	24	70.6	0.184
Heterozygous	12	31.6	6	17.6	
Homozygous for the Ins C allele	1	2.6	4	11.8	
*-141C* alleles					
Del C	37	74.0	30	75.0	0.914
Ins C	13	26.0	10	25.0	
*rs6280* genotypes					
Homozygous for the T allele	16	42.1	9	26.5	0.311
Heterozygous	13	34.2	17	50.0	
Homozygous for the C allele	9	23.7	8	23.5	
*rs6280* alleles					
C	45	40.8	25	41.7	0.551
T	29	59.2	35	58.3	

*Metabolic Syndrome †χ² Test; level of significance: p < 0.05.

There was no association considering the three components of MS, high blood pressure, central obesity and hypertriglyceridemia according to the polymorphisms of interest in this study. Low HDL showed a significant association with homozygous genotype for the Ins C allele of the *-141C* polymorphism of the *DRD2* gene (p < 0.05), as described in Table 3.

**Table 3:** Analysis of association of polymorphisms according to the metabolic syndrome component - Low HDL* (n = 72). Extended Western Region of Minas Gerais, MG, Brazil, 2015

Polymorphism	Without low HDL*		With low HDL*		p†
	N	%	N	%	
*Taq*IA genotypes					
Homozygous for the C allele	30	52.6	7	46.7	0.901
Heterozygous	23	40.4	7	46.7	
Homozygous for the T allele	4	7.0	1	6.7	
*Taq*IA alleles					
W	83	72.8	21	70.0	0.819
T	31	27.2	9	30.0	
-*141C* genotypes					
Homozygous for the Del C allele	38	66.7	11	73.3	0.032
Heterozygous	17	29.8	1	6.7	
Homozygous for the Ins C allele	2	3.5	3	20.0	
-*141C* alleles					
Del C	93	81.6	23	76.7	0.545
Ins C	21	18.4	7	23.3	
*rs6280* genotypes					
Homozygous for the T allele	20	35.1	5	33.3	0.952
Heterozygous	24	42.1	6	40.0	
Homozygous for the C allele	13	22.8	4	26.7	
*rs6280* alleles					
W	50	43.9	14	46.7	0.874
T	64	56.1	16	53.3	

*High-Density Lipoprotein †χ² Test; level of significance: p < 0.05.

As for hyperglycemia, the only polymorphism that presented a statistically significant association was the *rs6280* of the *DRD3* gene, in which the homozygous genotype for the T allele had a higher prevalence and was associated with non-hyperglycemia, as shown in the table below (Table 4).

**Table 4:** Analysis of association of polymorphisms according to the metabolic syndrome component - Hyperglycemia (n = 72). Extended Wester Region of Minas Gerais, MG, Brazil, 2015

Polymorphism	Without Hyperglycemia		With Hyperglycemia		p^*^
	N	%	N	%	
*Taq*IA genotypes					
Homozygous for C allele	15	62.5	22	45.8	0.084
Heterozygous	6	25.0	24	50.0	
Homozygous for T allele	3	12.5	2	4.2	
*Taq*IA alleles					
W	36	75.0	68	70.8	0.554
T	12	25.0	28	29.2	
-*141C* genotypes					
Homozygous for C allele	15	62.5	34	70.8	0.098
Heterozygous	9	37.5	9	18.8	
Homozygous for Ins C allele	0	0	5	10.4	
-*141C* alleles					
Del C	39	81.3	77	80.2	0.776
Ins C	9	18.8	19	19.8	
*rs6280* genotypes					
Homozygous for T allele	13	54.2	12	25.0	0.040
Heterozygous	6	25.0	24	50.0	
Homozygous for C allele	5	20.8	12	25.0	
*rs6280* alleles					
W	16	33.3	48	50.0	0.021
T	32	66.7	48	50.0	

*χ² Test; significance level: p <0.05.

The result of the analysis of the factor associated with the *-141C* variant of the *DRD2* and *rs6280* genes, component of the syndrome, low HDL, is shown in Table 5. The homozygous genotype for the Ins C allele of the *-141C* polymorphism (OR: 19.8) was associated with low HDL, that is, in this study, it was evidenced that a patient diagnosed with refractory schizophrenia who presented the *-141C* variant of the *DRD2* gene and had the homozygous genotype for the Ins C allele was almost 20-fold more likely to present a low HDL level when compared to the heterozygous genotype.

**Table 5:** Odds Ratio Calculation* (OR) with respective 95%Confidence Interval (95% CI). Extended Western Region of Minas Gerais, MG, Brazil, 2015

	OR*	CI 95%^†^ for OR	
		Low limit	Upper limit
*-141C* polymorphism	Low HDL		
Genotypes			
Heterozygous	1.00	-	-
Homozygous for the Ins C allele	19.8	1.51	701.1

* Odds Ratio †95% CI - 95% Confidence Interval.

## Discussion

This investigation brings as one result the non-association of the *Taq*IA, -*141C* and *rs6280* polymorphisms of the dopaminergic system with MS. However, it is a very relevant investigation because it showed the components that can lead to MS. In addition, a better elucidation of these factors is important to avoid other disastrous complications in the health of these patients^14^.

Metabolic disturbances are more prevalent in schizophrenic patients than in the general population and this may translate into an increased risk of chronic degenerative diseases and even mortality in this group^14^. According to Papanastasiou (2013), women tend to have increased rates of MS compared to men, a datum corroborated in the present study. However, that data must be carefully interpreted; men are likely to be at increased risk of developing cardiovascular disease as the possible result of a combination of unhealthy lifestyles and lack of health care when compared to women^15^ ^-^ ^16^.

The present study revealed a statistically significant difference in the genotypic distribution of the SNP *-141C* Ins/Del of the *DRD2* gene, with association of the homozygous Ins C genotype when analyzed with the low HDL levels. Some studies that have investigated the association of this polymorphism with schizophrenia found an increased frequency of this allele (Ins) in this group of patients^17^ ^-^ ^18^. A study conducted in 2010 found that significant weight gain was confirmed after 6 weeks of treatment with risperidone or olanzapine in the population of patients with the *-141C* Del allele. ^19^. In the present investigation, the Del C allele was also observed to have the highest frequency.

An investigation that sought to characterize the metabolic profile of patients who presented the first episode of schizophrenic outbreak reported a higher prevalence of low HDL in this group than the general population, but there was no association with the genetic profile of this population^20^. Low HDL levels predict increased cardiovascular risk, while high HDL levels are associated with a drop of up to 40% in cardiovascular risk compared to low HDL alone; that is to say, when HDL levels are below the recommended levels, there is risk of complications, especially of cardiac nature^21^ ^-^ ^22^.

There are studies that provide evidence of the association of altered glycemic levels with schizophrenia, but no association with the *rs6280* polymorphism of the *DRD3* gene has been found in the literature^8^ ^-^ ^23^. A study that analyzed the use of conventional and atypical antipsychotics in schizophrenic patients and correlated this factor with MS and its components showed that clozapine was the second most potent drug in the induction of MS. It also demonstrated that this drug was related with weight gain in patients after 16 weeks of continuous treatment, together with a significant increase in glycemic and lipid parameters^24^.

The change in glycemic levels in this particular group of patients may have even more harmful repercussions because the disease may induce metabolic alterations and some drug treatments may potentiate this risk. It is therefore necessary to monitor glycemic levels in all patients diagnosed with refractory schizophrenia in order to reduce morbidity and mortality risks. The present study found the association of the genotype of this variant *rs6280* to absence of hyperglycemia, and this suggests that the presence of this variant, especially in those who present homozygosis for the T allele, is a protective factor against hyperglycemia.

The monitoring of this metabolic alteration is fundamental for the evaluation of cardiovascular risk. The imbalance of this marker is an indicator of risk for chronic-degenerative diseases and increased mortality. Routine follow-up on a regular basis and education on healthy lifestyle are necessary to minimize the risks of complications^2^ ^-^ ^15^. The hypotheses raised in this study suggest that the health care for refractory schizophrenic patients using clozapine must be comprehensive, and the planning of strategies aimed at minimizing the risk of metabolic alterations should be one of the main goals of the individual therapeutic plan for these patients^2^ ^-^ ^14^.

The findings of this study indicated relevant issues when highlighted the vulnerability to which refractory schizophrenic patients are exposed. Metabolic changes increase the risk for the development of cardiovascular diseases and other health implications, which directly interfere with the quality of life of these patients. Therefore, it is essential to develop planned actions and periodic monitoring of plasma levels of biochemical markers that may lead to MS in order to prevent metabolic alterations.

In this sense, it is important that professionals directly involved in the care of these patients know their different metabolic profiles and the possible adverse effects to which they are exposed, so as to conduct the appropriate treatment for each of them. Awareness of the possibility of these effects must also be raised among patients, allied to promotion of healthy habits, especially regarding a healthy diet and the practice of physical activity.

The limitations of this study are related to its external validity, considering that the sample was not probabilistic and caution is necessary regarding the generalization of the results. The research design allowed estimating the prevalence of the polymorphisms as well as the associated factors, and it is not possible, therefore, to make cause-effect inferences. In addition, we suggest the development of longitudinal researches to improve the understanding of aspects pertinent to the development of MS, establishing associations with the polymorphisms of interest. However, the chosen design provided satisfactory responses to the guiding questions and objectives of the study.

## Conclusion

The results of this study indicated that the prevalence of MS in people with refractory schizophrenia using clozapine is high, but there was no association with the polymorphisms of interest. The ancestral genotype appeared to be a protective factor against hyperglycemia in this sample. Therefore, these findings point to the need to carry out further studies involving genetic analyses and MS in patients with refractory schizophrenia in order to guide the drug treatment and promote a better quality of life of this clientele.
